# Mono-ADP-ribosylating PARP enzymes in cellular signaling and disease

**DOI:** 10.1242/jcs.263963

**Published:** 2025-07-31

**Authors:** Simone Di Paola, Giovanna Grimaldi, Daniela Corda

**Affiliations:** Institute of Endotypes in Oncology, Metabolism, and Immunology ‘G. Salvatore’ (IEOMI), National Research Council (CNR), Via Pietro Castellino 111, 80131, Naples, Italy

**Keywords:** Mono-ADP-ribosylation, PARPs, Cellular Signaling, Disease, Cancer

## Abstract

ADP-ribosyl-transferases (ARTs) are versatile post-translational regulators. Mammalian ARTs include poly- and mono-ADP-ribosylating enzymes, which transfer ADP-ribose molecules deriving from β-NAD^+^ to their targets. Mono-ADP-ribosylation (MARylation), which is catalyzed by mono-ARTs such as PARP3, PARP6–PARP12 and PARP14–PARP16, tunes the activity of targets involved in fundamental cell processes and various signaling pathways, ranging from those regulating cell survival and proliferation to those modulating the cellular response to stress and viral infection. Recent advancements of techniques that enable the discovery of MARylation targets across cellular compartments have further expanded our knowledge about the physiological roles of these targets and the potential connection between MARylation and the onset of pathologies. Furthermore, increasing efforts in the development of specific drugs targeting the different MARylating PARP proteins are opening avenues for innovative pharmacological treatments. In this Review, we illustrate the cell cycle progression, intracellular membrane trafficking and cellular stress pathways regulated by mono-ART PARP proteins. We then describe what is known about the roles of MARylating PARP proteins in the context of viral infection and cancer. Finally, we discuss potential future directions towards mapping out the complex network of PARP targets and functions.

## Teaser

**Summary:** We discuss the roles of mono-ADP-ribosylation catalyzed by PARP enzymes in basic cell functions, including the cell cycle, DNA repair, trafficking and stress responses, as well as in viral infections and cancer.

## Introduction

**Fig. 1.**
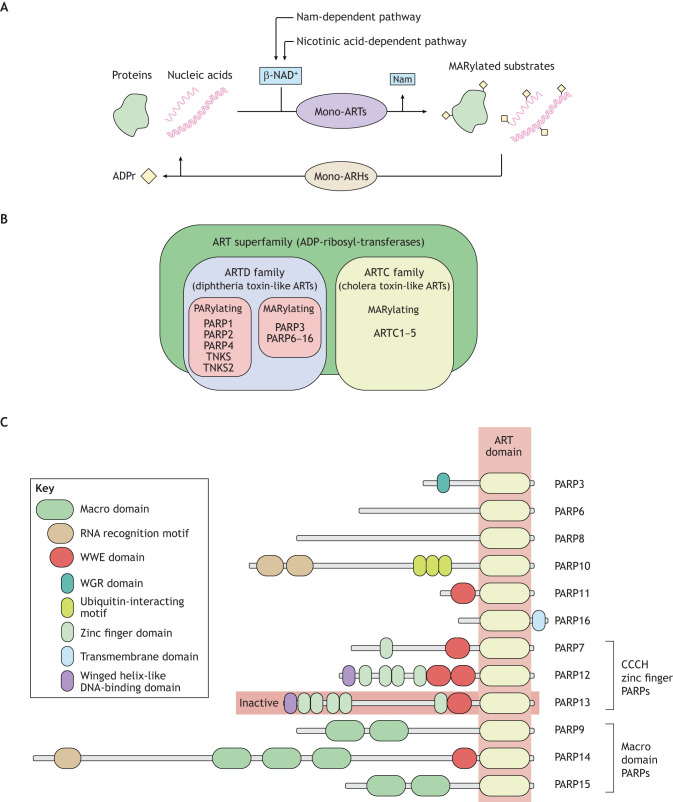
**The MARylation reaction and MARylating PARPs.** (A) MARylation is a reversible process involving the cooperative action of the mono-ARTs and enzymes of the glycohydrolase family (mono-ARHs). Mono-ARTs catalyze the transfer of an ADPr molecule from β-NAD^+^ to proteins or nucleic acids, with the concomitant release of a nicotinamide (Nam) molecule. Mono-ARHs catalyze the reversal of this reaction through the hydrolysis of the bonds between the MARylated substrates and ADPr, generating free ADPr. (B) Schematic representation of ART enzyme categorization and nomenclature. The ART superfamily comprises diphtheria toxin-line ART (ARTD) and cholera toxin-like ART (ARTC) families. The ARTD family includes PARPs with both MARylating activity (PARP3, PARP6–PARP16) and PARylating activity (PARP1, PARP2 and PARP4), as well as TNKS and TNKS2 PARylating enzymes. The ARTC family contains the MARylating enzymes ARTC1–ARTC5. (C) MARylating enzymes belonging to the PARP family include PARP3 and PARP6–PARP16, with PARP13 being enzymatically inactive. The MARylating PARPs are characterized by a common catalytic ART domain flanked by varying domains that confer specificity of interactions, such as domains involved in protein binding (e.g. ubiquitin-interacting motifs), nucleic acid binding (e.g. WGR domains, RNA recognition motifs, zinc finger domains and winged helix-like domains) and ADPr binding (e.g. WWE domains, Macro domains).

ADP-ribosylation is an ancient protein post-translational modification (PTM), catalyzed by ADP-ribosyl-transferases (ARTs) (Fig. 1A). A diversification of ARTs occurred during the evolution of bacterial conflict systems (offensive and defensive mechanisms that originated to compete with other organisms for resources and survival), such as virus–host interactions and toxin–antitoxin systems. ADP-ribosylation was originally discovered as the pathogenic mechanism of action of diphtheria toxin (Collier and Pappenheimer, 1964). Numerous additional toxins were subsequently reported to possess ART activities, which are now established as toxic mechanisms employed by many bacteria (Grimaldi et al., 2015; Holbourn et al., 2006). Based on this knowledge, the search for mammalian ARTs flourished and led to the identification of enzymes catalyzing poly- and mono-ADP-ribosylation (PARylation and MARylation, respectively) of cellular substrates, raising interest in the physiological role of these PTMs (Corda and Di Girolamo, 2003; Feijs-Žaja et al., 2024). In eukaryotic evolution, ARTs were repeatedly acquired via lateral transfer, and their functions diversified accordingly (Aravind et al., 2015). The ART superfamily comprises the cholera toxin-like ART (ARTC) and diphtheria toxin-like ART (ARTD) families (Fig. 1B). The ARTD family includes members that catalyze both PARylation and MARylation. In this Review, we will focus on the functions of mono-ARTs of the ARTD family; it is important to note that although these enzymes are denoted as poly-ADP-ribose polymerases (PARPs), they add only one ADP-ribose to their substrates (Lüscher et al., 2022b) (Fig. 1C). For a brief overview of PARylating ARTs, see Box 1.

Box 1. Poly-ARTs, non-PARP mono-ARTs and hydrolases The accepted nomenclature of the ART superfamily (Lüscher et al., 2022a,b) includes the ARTD and ARTC families (Fig. 1B). The ARTD family comprises PARP1–PARP4, PARP6–PARP16, TNKS (also known as TNKS1 and PARP5a) and TNKS2 (also known as PARP5b). The ARTC family comprises ARTC1–ARTC5 (also known as ART1–ART5).

PARP1 and PARP2, which share structural and functional homology, catalyze PARylation, the addition of long, sometimes branched, polymeric chains of ADPr to protein substrates, (Jessop et al., 2024). PARylation plays a central role in the response to DNA damage by facilitating the recruitment of effector proteins to damage sites and promoting single- and double-strand DNA break repair. PARylation also regulates chromatin organization and gene transcription through mechanisms that require rapid and localized control of genomic structure (Azarm and Smith, 2020; Jessop et al., 2024; Suskiewicz et al., 2020). Despite similarities to PARP1, PARP2 also has unique roles in inflammation and cancer (reviewed in Szántó et al., 2024). Additional poly-ARTs include PARP4, as well as TNKS and TNKS2, which participate in, among other processes, telomere length homeostasis, mitosis and Wnt–β-catenin signaling (reviewed in Jessop et al., 2024). TNKS and TNKS2 are also promising drug targets, but the activity of specific inhibitors is often hampered by localized on-target toxicity (Lau et al., 2013). Recently, a small molecule, STP1002, has been reported as efficacious for colon cancer treatment in preclinical studies, without local toxic effects (Kim et al., 2022).

PARylation can be removed by the poly-ADP-ribose glycohydrolase PARG, which hydrolyzes the glycosidic bond linking the ADPr polymers; however, PARG cannot act on the final ADPr linked to the protein (Rack et al., 2020)**.** A different family of glycohydrolases, the ADP-ribosylhydrolases (ARH1, ARH2 and ARH3, also known as ADPRH, ADPRHL1 and ADPRS, respectively), act specifically on MARylated arginine and serine residues (Bartlett et al., 2018; Rack et al., 2020). The MARylation of these residues is catalyzed by ARTC proteins (also called ecto-ARTs) (Hottiger et al., 2010). The five ARTC members are glycosylphosphatidylinositol (GPI)-anchored proteins that either face the extracellular or the intraluminal space or are secreted. ARTCs are involved in the regulation of immune functions, signaling and the ER stress response (Fabrizio et al., 2015; Seman et al., 2003), or are inactive, as in the case of ARTC3 and ARTC4 (Di Girolamo and Fabrizio, 2019; Menzel et al., 2018). Other enzymes able to reverse the ADP-ribosylation reaction are phosphodiesterases of the nucleoside diphosphates linked to other moieties ‘X’ (NUDIX) and ectonucleotide pyrophosphatase/phosphodiesterase (ENPP) families, which cleave the pyrophosphate bond, producing a ribose-5′-phosphate tag on the substrate (Borza et al., 2022; McLennan, 2006).

MARylation involves the transfer of a single ADP-ribose unit from β-NAD^+^ onto specific residues of protein substrates. This reaction, catalyzed by specific mono-ARTs including PARP3 and PARP6–PARP16 (with PARP13, which is encoded by *ZC3HAV1*, being enzymatically inactive; PARP7 is also known as TiPARP) (Fig. 1C), is involved in a variety of biological processes, including the regulation of cell signaling, intracellular membrane trafficking, metabolism, stress responses, viral infection, immune responses and tumorigenesis (Corda and Di Girolamo, 2003; Lüscher et al., 2022b; Morone and Grimaldi, 2024; Suskiewicz et al., 2023). ADP-ribosylation is a highly dynamic process supported by the cooperative action of ARTs and enzymes of the glycohydrolase family (Box 1) that reverse the reaction through the hydrolysis of the bonds between the modified protein residues and adenosine diphosphate ribose (ADPr) (Fig. 1A). These glycohydrolase enzymes are localized in different cell compartments and bear specificity for the type of bond and acceptor residues (reviewed in Ishiwata-Endo et al., 2022; Stevens et al., 2019). A subset of Macro domain-containing hydrolases, including MacroD1, MacroD2 and TARG1 (also known as OARD1), act only on single ADPr units linked by ester bonds to acidic amino acids. These enzymes can be localized in mitochondria (MacroD1), in the cytosol and nucleus (MacroD2), or in the nucleus and stress granules (TARG1) (Žaja et al., 2020).

Recently, DNA and RNA have been found to be additional and somewhat unexpected substrates of mono-ARTs (Fig. 1A). PARP1- and PARP3-dependent DNA MARylation at sites of DNA breaks was first reported *in vitro* (Munnur and Ahel, 2017; Zarkovic et al., 2018). Recently, RNA MARylation has been demonstrated in mammalian cells, catalyzed by mono-ARTs such as TRPT1, PARP10, PARP11, PARP12 and PARP15 (Weixler et al., 2022). The reverse reaction involves MacroD1 or MacroD2 and TARG1, which remove ADPr from both DNA and RNA (Munnur and Ahel, 2017; Munnur et al., 2019; Weixler et al., 2022). Although we will not cover these DNA and RNA modifications here, they represent an active field of investigation that will undoubtedly expand the physiological relevance of mono-ART activities (Munnur and Ahel, 2021; Weixler et al., 2022). For more details on the emerging roles of nucleic acid MARylation, we refer readers to recent reviews (Munnur and Ahel, 2021; Weixler et al., 2021).

Many recent, detailed reviews and reports have comprehensively addressed the components that determine and reverse the ADP-ribosylation reaction. Below, we will instead review mechanistic aspects of the MARylating PARPs within the physiological processes they regulate as well as in pathological contexts. Alterations in MARylation are often associated with pathologies such as cancer or viral infections, making ADP-ribosylation a promising target for the development of new therapeutics.

## MARylating PARPs in cell cycle regulation, DNA replication stress and the DNA damage response

**Fig. 2.**
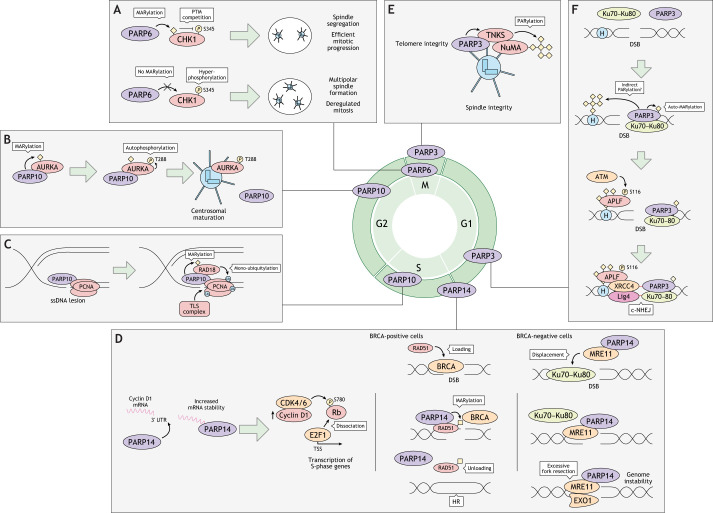
**MARylating PARPs in cell cycle regulation, DNA replication stress and the DDR.** PARP3, PARP6, PARP10 and PARP14 play crucial roles in cell cycle progression and genome stability. (A) PARP6 regulates mitosis by MARylating CHK1, preventing centrosome amplification and multipolar spindle formation. (B,C) PARP10 activates AURKA during G2 phase, ensuring proper centrosome maturation and mitotic entry. PARP10 also alleviates replication stress in S phase by facilitating TLS through interactions with RAD18 and PCNA. (D) PARP14 promotes the G1-S transition by stabilizing cyclin D1 mRNA. In BRCA-positive cells, PARP14 enhances HR by regulating RAD51, whereas in BRCA negative cells, PARP14 recruits MRE11 to stalled replication forks, promoting fork restart. (E,F) PARP3 maintains spindle integrity during mitosis by interacting with NuMA and TNKS, and also facilitates NHEJ by MARylating Ku70–Ku80, promoting DNA repair. BRCA, BRCA1 and BRCA2; CDK4/6, CDK4 or CDK6; c-NHEJ, canonical NHEJ; H, histone; *P*, phosphorylation; TSS, transcription start site; Ub, ubiquitin.

MARylation catalyzed by PARPs plays an important role in transcriptional and post-translational regulation of proteins involved in cell cycle signaling pathways, which are key determinants of cell proliferation. Several PARPs have been identified as regulators of different aspects of the cell cycle: PARP3, PARP6, PARP10 and PARP14 are involved in mitotic spindle function, cell cycle phase transitions and genome stability during DNA replication. The latter function overlaps with a broader role of some of these enzymes as genome caretakers during the DNA damage response (DDR) to genotoxic stress (Fig. 2A–F).

PARP6 and PARP10 are soluble enzymes that modify phase-specific kinases critical for the progression of the cell cycle. During mitosis, PARP6 MARylates checkpoint kinase 1 (CHK1, also known as CHEK1), a serine/threonine protein kinase, modulating its activity by negatively regulating phosphorylation of CHK1 at Ser345. CHK1 plays a crucial role in the DDR by blocking the G2-M transition and regulating checkpoints in S and M phases. Inhibition of PARP6 leads to hyperactivated CHK1, which is associated with amplification and clustering of centrosomes, resulting in multipolar spindles (Fig. 2A) (Wang et al., 2018). Furthermore, the G2 kinase Aurora A (AURKA) is a well-established substrate of PARP10 (Di Paola et al., 2022; Feijs et al., 2013; Zhao et al., 2018). Following its transcriptional upregulation during G2 phase, AURKA primarily concentrates in centrosomes, where it promotes centrosome maturation and mitotic spindle assembly through phosphorylation of various substrates, facilitating the G2-M transition (Nikonova et al., 2013). MARylation of AURKA by PARP10 stimulates AURKA activation by enhancing autophosphorylation on Thr288, and depletion or inhibition of PARP10 disrupts the timely recruitment of AURKA to the centrosomes and mitotic spindle during G2, leading to delayed mitotic entry (Fig. 2B) (Di Paola et al., 2022).

PARP14 regulates the G1-S transition by increasing the stability of cyclin D1 (CCND1) mRNA through binding to its 3′ untranslated region (UTR), most likely via RNA recognition motifs. Transient depletion of PARP14 causes a decrease in cyclin D1 expression and a concomitant reduction in phosphorylation of retinoblastoma (Rb, also known as RB1) by CDK4 or CDK6 that is necessary for the transcription of S-phase genes by the transcription factor E2F1, leading to the accumulation of cells in G1 phase (Fig. 2D) (O′Connor et al., 2021).

PARP3, a nuclear enzyme that localizes at the centrosomes, participates in the maintenance of spindle integrity during mitosis. At centrosomes, PARP3 binds the spindle components nuclear mitotic apparatus (NuMA, also known as NUMA1) and tankyrase (TNKS), stimulating PARylation of NuMA by TNKS. Ablation of PARP3 causes accumulation of abnormal mitotic figures displaying an aberrant metaphase configuration, metaphase arrest and telomere fusion (Fig. 2E) (Boehler et al., 2011).

During S phase, slowing or stalling of replication fork progression and/or DNA synthesis, defined as replication stress, can occur (Zeman and Cimprich, 2014). Replication stress is mainly characterized by persistent single-stranded DNA (ssDNA) structures that must be carefully managed to prevent the accumulation of damage during DNA synthesis. PARP10 actively participates in S-phase DNA repair to alleviate replication stress via stimulation of translesion synthesis (TLS), a DNA damage tolerance pathway that enables the replication machinery to bypass DNA lesions, restarting replication (Khatib et al., 2024b). Under conditions causing damage-induced gaps in DNA, catalytically active PARP10 localizes to nascent DNA and interacts with RAD18, the main ubiquitin ligase responsible for the ubiquitylation of proliferating cell nuclear antigen (PCNA), another PARP10 interactor (Khatib et al., 2024a; Nicolae et al., 2014). PCNA ubiquitylation in turn recruits the TLS polymerase REV1 to fill gaps (Fig. 2C). Thus, PARP10 allows restarting of stalled replication forks, alleviating replication stress and promoting cellular proliferation (Schleicher et al., 2018).

PARP14 contributes to genomic stability through the regulation of homologous recombination (HR), an error-free pathway that is activated upon detection of DNA double-strand breaks (DSBs) (Fig. 2D) (Dhoonmoon and Nicolae, 2023). In HR, the breast cancer susceptibility proteins BRCA1 and BRCA2 facilitate the loading of RAD51 onto ssDNA at the damage site, forming right-handed helical filaments on ssDNA. This nucleoprotein scaffold complex is essential for searching, synapsing with and invading the homologous DNA sequence on the sister chromatid (Wright et al., 2018). In cells expressing BRCA1 and BRCA2, PARP14-mediated MARylation of RAD51 promotes its unloading from DNA, thereby enhancing HR efficiency (Nicolae et al., 2015). In cells negative for BRCA1 or BRCA2, PARP14 recruits the nuclease MRE11 to stalled replication forks bound by the Ku70–Ku80 (XRCC6–XRCC5) heterodimer. MRE11 removes Ku70–Ku80, allowing the 5′–3′ exonuclease EXO1 to access the DNA and catalyze long-range resection (Dhoonmoon et al., 2022). In certain cases, HR is also involved in alleviating replication stress, and PARP14 might additionally be involved in these pathways. Disruption of the kinases ATR and CHK1, which regulate the replication stress response, has been found to decrease cell viability in PARP14-deficient cells owing to defective replication and cell cycle checkpoint failure, potentially leading to mitotic catastrophe (Dhoonmoon et al., 2020).

PARP3 takes part in non-homologous end joining (NHEJ), the major pathway for repairing DSBs, by interacting with and MARylating Ku70–Ku80, thereby facilitating binding and protection of DNA ends at the damaged site (Fig. 2F) (Beck et al., 2014). In parallel, PARP3-mediated MARylation of histones favors the accumulation of aprataxin and PNKP-like factor (APLF) at the damage site through the PBZ domain of APLF, and APLF localized to DNA is phosphorylated on Ser116 by ATM in a PARP3-dependent manner, increasing its association with DNA (Fenton et al., 2013). This might favor the recruitment of additional NHEJ factors like XRCC1 and Lig4, accelerating DNA ligation and completion of NHEJ (Rulten et al., 2011).

## MARylating PARPs in protein trafficking and cellular stress responses

**Fig. 3.**
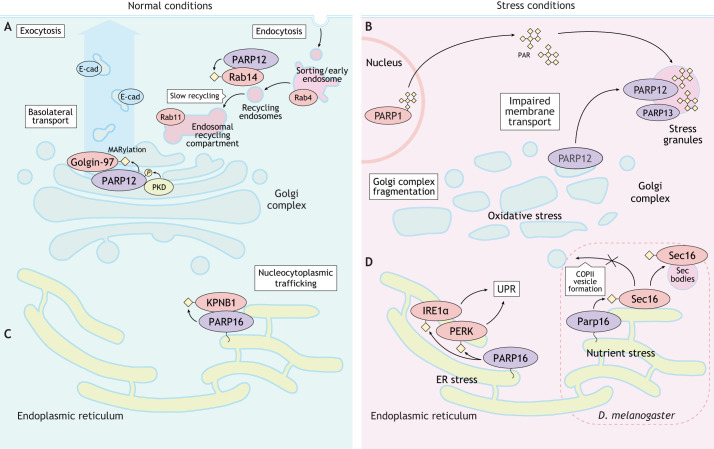
**MARylating PARPs in protein trafficking and cellular stress responses.** MARylating PARPs adapt their functions to restore cellular homeostasis under stress conditions. (A) Under normal conditions, PARP12 is primarily localized at the Golgi, where it regulates intracellular membrane trafficking by modifying Golgin-97, which plays a key role in transporting basolateral cargoes like E-cad that are essential for cell polarity and adherens junction formation. Additionally, PARP12 modifies Rab14, a GTPase involved in vesicular trafficking, facilitating endocytic recycling. *P*, phosphorylation; PKD, protein kinase D. (B) PARP12 relocates to stress granules under oxidative stress, leading to Golgi fragmentation and a reversible halt in anterograde membrane trafficking. This is driven by nuclear PAR formation, which translocates to the cytosol and acts as a scaffold for stress granule assembly. (C) Under normal conditions, PARP16 is anchored to the ER where it MARylates importin-β1 (KPNB1), a protein involved in nucleocytoplasmic trafficking. (D) Upon ER stress, PARP16 MARylates PERK and IRE1α, two kinases that are central to the UPR, enhancing their activity and downstream signaling. In *D. melanogaster*, Parp16 modifies Sec16 under nutritional stress, impairing the secretory pathway by inducing formation of Sec bodies.

Eukaryotic cells are exposed to many stresses arising from different sources. In order to re-establish physiological conditions following stress, they have consequently developed the capability to modify their basal functions by activating specific signaling pathways. Some PARPs participate in pathways that are activated in response to different types of stress. In most cases, their cellular localization is indicative of the functions that these enzymes play in cellular physiology and stress response (Fig. 3A–D).

The mono-ART PARP12 is localized at the Golgi (Vyas et al., 2013) and is involved in the control of intracellular membrane trafficking. Under oxidative stress, translocation of PARP12 from the Golgi to stress granules is associated with Golgi fragmentation and a reversible block in anterograde membrane trafficking (Fig. 3A,B) (Catara et al., 2017). This process is driven by the PARP1-dependent formation of nuclear poly-ADP-ribose (PAR), which translocates to the cytosol and forms a scaffold for the assembly of stress granules. PARP12-dependent blockage of anterograde membrane trafficking can be prevented under stress conditions by inhibiting PARP1 enzymatic activity via olaparib treatment or by PARP1 knockdown (Catara et al., 2017; Grimaldi and Corda, 2019). Thus, PAR and/or PARylated proteins act as signaling molecules that mediate the interplay between nuclear and cytoplasmic PARP activities, highlighting the functional interaction between PARP1 and PARP12 in cellular stress responses (Grimaldi and Corda, 2019). This cross talk might possibly intervene in other PARP-related functions (Fig. 3B).

These observations have led to in-depth analysis of the regulation of intracellular membrane trafficking by PARP12. Golgin-97 (also known as GOLGA1) – a tethering protein crucial in the regulation of the transport of specific basolateral cargoes, such as E-cadherin (E-cad, also known as CDH1), from the trans-Golgi network (TGN) to the plasma membrane – has been identified as a specific substrate of PARP12 (Grimaldi et al., 2022), with MARylation of golgin-97 required for its function. This modification facilitates the formation and release of tubulovesicular carriers from the TGN, ensuring efficient delivery of E-cad to the plasma membrane, a process that is essential for maintenance of cell polarity and the formation of adherens junctions in epithelial cells. Defects in this process are involved in cancer cell spreading (Fig. 3A) (Janiszewska et al., 2020).

PARP12 also functions in the endocytic recycling pathway. Rab14, a small GTPase involved in vesicular trafficking, undergoes PARP12-mediated MARylation at specific glutamic acid residues (Lo Monte et al., 2018). This modification facilitates endosomal progression through specific Rab4-, Rab14- and Rab11-positive compartments, directing vesicles toward the endocytic recycling route and ultimately to the plasma membrane (Fig. 3A) (Corteggio et al., 2022 preprint). Our group has recently found evidence that additional Rab family members are substrates of PARP-dependent MARylation (Grimaldi et al., 2024), pointing to multiple roles that mono-ARTs might play in membrane trafficking as well as in the other cellular functions discussed in this Review.

PARP16 is the sole transmembrane mono-ART belonging to the PARP family and is anchored to the endoplasmic reticulum (ER) membrane via a single transmembrane tract, with its mono-ART domain facing the cytoplasm (Di Paola et al., 2012). Initial PARP16 overexpression studies showed that PARP16 binds and modifies importin-β1 (KPNB1), a nuclear transport chaperone, suggesting that MARylation of this soluble factor might play a role in the regulation of nucleocytoplasmic protein trafficking or in KPNB1 ER-related functions, such as ER-associated degradation (ERAD) (Fig. 3C) (Di Girolamo, 2015).

Importantly, PARP16 is part of the signaling network that is activated in response to ER stress, the unfolded protein response (UPR) (Fig. 3D) (Hetz et al., 2020; Jwa and Chang, 2012). The UPR comprises three main branches: two branches relying on the transmembrane kinases PERK (also known as EIF2AK3) and IRE1α (also known as ERN1), and a third dependent on activating transcription factor 6 (ATF6). ER stress activates PARP16, which MARylates PERK and IRE1α, stimulating their trans-phosphorylation and the activation of specific signaling cascades and transcriptional responses (Jwa and Chang, 2012). In line with this role, silencing of PARP16 decreases the transcription of known UPR-dependent genes (Jwa and Chang, 2012). Interestingly, the ER-luminal C-terminal tail of PARP16 plays a crucial role in stimulating its cytosolic mono-ART activity. Mutation of the 23-amino-acid tail impairs MARylation of PERK and IRE1α upon ER stress, suggesting that the tail is required for PARP16 to sense ER luminal stress and/or facilitate the recruitment of its substrates (Jwa and Chang, 2012). In *Drosophila melanogaster*, nutrient stress induced by amino acid starvation stimulates Parp16-mediated MARylation of Sec16, a protein involved in the formation and budding of COPII-coated vesicles from ER exit sites (ERES) (Aguilera-Gomez et al., 2016). Together with other components of the COPII machinery, MARylated Sec16 translocates from ERES and coalesces into condensates, termed Sec bodies, which impair the secretory pathway (Fig. 3D).

Another PARP with a potential role in cellular stress responses is PARP8, a cytosolic mono-ART enriched at the nuclear envelope during interphase, which translocates to the spindle poles during mitosis. Using activated ion electron transfer dissociation mass spectrometry, auto-modification of four cysteine residues (Cys332, Cys367, Cys376 and Cys395) of PARP8 (Vyas et al., 2013, 2014) has been confirmed. Cys332, Cys376 and Cys395 modifications occur under basal conditions, whereas C367 is modified only following H_2_O_2_ stress (Buch-Larsen et al., 2020), hinting at a potential role of PARP8 in H_2_O_2_ stress response (Vyas et al., 2014). Similarly, under stress conditions, PARP15 is localized at stress granules together with four other PARPs (TNKS, PARP12, and the PARP13 isoforms PARP13.1 and PARP13.2). PARP15 binds PAR via its Macro domain and might be involved in modification of the enzymatically inactive PARP13, potentially altering the interactions with its molecular partners (Leung et al., 2011).

## MARylating PARPs in viral infections

**Fig. 4.**
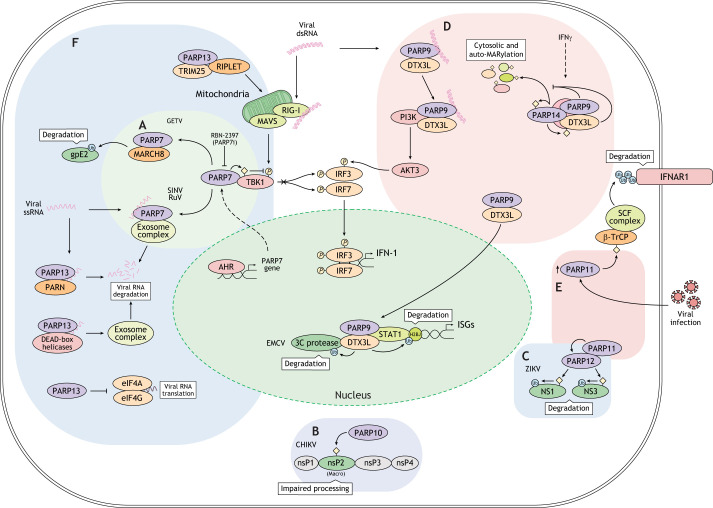
**MARylating PARPs in viral infections.** Several MARylating PARPs play key roles in antiviral defense. (A–C) PARP7 suppresses IFN-I signaling by MARylating the kinase TBK1, thereby downregulating the innate immune response. Additionally, PARP7, along with PARP10 and PARP12 (in collaboration with PARP11), can degrade viral RNA, inhibit translation or stimulate ubiquitin-mediated degradation of viral proteins. (D) PARP9, in complex with DTX3L, contributes to viral protease degradation and regulation of ISGs. PARP14 modulates IFN-I responses in infections caused by coronaviruses and other viruses. (E) PARP11 modulates the levels of the IFN receptor, thus affecting the strength of IFN-I-activated signaling. (F) PARP13 selectively recognizes and degrades viral RNAs in cooperation with several different interactors. *P*, phosphorylation; PARP7i, PARP7 inhibitor; Ub, ubiquitin.

The innate immune response serves as the primary defense against invading viruses. During viral infection, pattern recognition receptors (PRRs) detect pathogen-associated molecular patterns (PAMPs) (Diebold et al., 2004; Oshiumi et al., 2016). Upon recognizing these patterns, host cells produce and release interferons (IFNs), which activate autocrine and paracrine signaling pathways. This activation induces the expression of interferon-stimulated genes (ISGs) and ultimately restricts the infection (Diebold et al., 2004; Oshiumi et al., 2016). Several MARylating PARPs (PARP7 and PARP9–PARP15) are induced by type I interferons (IFN-I) or PAMPs and, as part of the innate immune response, interfere with the replication of viruses either by regulating host innate immune signaling or by directly affecting viral replication (Fig. 4A–F) (Lüscher et al., 2022a). Here, we will focus on the known molecular mechanisms of MARylating PARPs in the antiviral response and discuss future perspectives, particularly regarding their potential as pharmacological targets.

PARP7 acts as a negative regulator of IFN-I signaling, a key pathway in the innate immune response against viral infections and tumors (see below). During viral infections, the IFN-I response is initiated by cytosolic nucleic acid sensors that detect DNA or RNA. These pathways converge on TANK-binding kinase 1 (TBK1) activation, which leads to phosphorylation and nuclear translocation of IRF3–IRF7 dimers, ultimately driving IFNB1 expression (Chathuranga et al., 2021). PARP7 suppresses IFN-I signaling by interacting with and MARylating TBK1, blocking induction of IFN-I (Yamada et al., 2016). Some transcription factors, such as the aryl-hydrocarbon receptor (AHR) (see below), upregulate PARP7 expression, thereby dampening the IFN-I response during viral infection. Supporting this, AHR-deficient cells and mice exhibit enhanced virus-induced IFN-β production and restricted viral replication (Yamada et al., 2016). Similarly, a recent study has demonstrated that the PARP7 inhibitor RBN-2397 enhances innate antiviral immunity in mice infected with vesicular stomatitis virus (VSV) by increasing TBK1 phosphorylation, which in turn boosts IFN responses and strengthens the host antiviral defense (Fig. 4A) (Du et al., 2024).

PARP7 also exhibits antiviral activity by promoting viral RNA degradation, although this effect appears to be virus dependent. Replication of Sindbis virus (SINV) and rubella virus (RuV) is enhanced in PARP7-depleted U373 human astrocyte cells and in PARP7-knockout mice; this has been shown to be due to the loss of PARP7 binding to viral RNA via its zinc finger domain, which recruits the exosome complex, inducing viral RNA degradation (Fig. 4A) (Kozaki et al., 2017). Additionally, PARP7 together with PARP10 and PARP12 has been shown to inhibit Venezuelan equine encephalitis virus (VEEV) replication by blocking protein translation. PARP7 also inhibits replication of Getah virus (GETV), a model for *Alphavirus*, by promoting the ubiquitylation and degradation of the viral glycoprotein E2 (gpE2) via the host E3 ubiquitin ligase MARCH8 (also known as MARCHF8; Fig. 4A) (Jiao et al., 2023). VEEV, SINV and GETV all belong to the *Alphavirus* genus within the *Togaviridae* family, highlighting a crucial role of PARP7 in countering viruses within the *Togaviridae* family.

PARP9 contributes to the antiviral immune response through its interaction with deltex E3 ubiquitin ligase 3L (DTX3L). The PARP9–DTX3L complex plays a crucial role in reducing replication of multiple viruses, such as encephalomyocarditis virus (EMCV), influenza A virus and SINV (Fig. 4D). In the nucleus, this complex interacts with the transcription factor STAT1 and ubiquitylates histone H2BJ (also known as H2BC11) to enhance ISG expression; in parallel, the complex promotes degradation of the viral 3C proteases, thereby modulating both IFN signaling and the intrinsic replication capacity of the virus (Zhang et al., 2015). The PARP9–DTX3L complex can also function as a non-canonical sensor for RNA viruses, triggering IFN-I production through the phosphoinositide 3-kinase (PI3K)–AKT3 pathway (Xing et al., 2021). Mechanistically, PARP9 utilizes its Macro domains to recognize viral double-stranded RNA (dsRNA) and interacts with the p85 subunit of PI3Ks to activate the downstream PI3K–AKT3 pathway, thereby limiting RNA virus infections (Fig. 4D) (Xing et al., 2021). Silencing or deleting PARP9 significantly reduces IFN-I production in response to dsRNA or RNA virus infections (Xing et al., 2021). More recently, the PARP9–DTX3L complex has also been shown to cooperate with PARP14 within cytoplasmic foci (see below) that are specifically induced by IFNγ stimulation. Together, these proteins modulate the MARylation of as-yet-unidentified targets localized in these foci that most likely contribute to IFNγ signaling (Fig. 4D) (Kar et al., 2024; Ribeiro et al., 2024).

PARP10 is one of the MARylating PARPs stimulated by IFNs (Heer et al., 2020) and, alongside PARP12, has been shown to interfere with replication of the chikungunya virus (CHIKV) (Krieg et al., 2023). Mechanistically, the non-structural CHIKV protein nsP2 serves as a MARylation substrate for IFN-stimulated PARPs, including PARP10. MARylation by PARP10 impairs nsP2 proteolytic activity, thereby preventing polyprotein processing and inhibiting viral replication (Fig. 4B) (Krieg et al., 2023). Interestingly, PARP10, along with PARP7 and PARP12, has also been reported to inhibit mouse hepatitis virus (MHV) replication by affecting host protein translation (Atasheva et al., 2014), suggesting a cooperative role for PARP10 among other PARPs in mediating antiviral effects.

PARP11 inhibits IFN-I-activated signaling by modulating the protein levels of the IFN receptor IFNAR1 through MARylation of the E3 ubiquitin ligase β-TrCP (also known as BTRC), which enhances the ubiquitylation and degradation of IFNAR1, weakening the IFN-I response (Fig. 4E). Viral infections cause upregulation of PARP11 expression, facilitating viral immune evasion through this MARylation-mediated mechanism. Rucaparib, a clinically available drug, targets PARP11 to stabilize IFNAR1, enhancing IFN-I signaling and bolstering the host antiviral response, thus offering a promising strategy for improving the efficacy of IFN-I signaling and antiviral defense (Guo et al., 2019).

PARP12 is highly expressed in immune cells, such as macrophages, and functions as an ISG that is crucial for antiviral immunity (Atasheva et al., 2012). PARP12 restricts the replication of numerous viruses, including VEEV, SINV, EMCV and Zika virus (ZIKV) (Li et al., 2018, 2021a), as well as coronaviruses (Atasheva et al., 2012; Kerr et al., 2023; Lee et al., 2021). As an antiviral factor, PARP12 inhibits host translation machinery, blocking viral protein synthesis and replication (Atasheva et al., 2014). Under stress conditions, PARP12 localizes at cytosolic stress granules (Catara et al., 2017; Leung et al., 2011), where it can bind viral RNA and facilitate its degradation. In addition, PARP12 mediates the MARylation of viral proteins, such as ZIKV non-structural proteins NS1 and NS3, promoting their ubiquitylation and proteasomal degradation, a process that is enhanced by cooperation with PARP11 (Fig. 4C) (Li et al., 2021a). Recent findings further support the role of PARP12 in immune defense signaling pathways and restriction of viral replication. PARP12, as well as PARP7, directly MARylates the alphaviral non-structural proteins nsP3 and nsP4, thereby inhibiting viral replication (Ryan et al., 2025). Moreover, knockdown of zinc finger domain-containing PARPs (PARP12, PARP7 and PARP13) weakens the antiviral effects of IFNγ on alphavirus replication, underscoring the crucial role of PARP12 in both viral restriction and host immune defense (Ryan et al., 2025).

PARP13, which is also known as zinc finger antiviral protein (ZAP) or zinc finger CCCH-type antiviral protein 1 (ZC3HAV1), is a potent stimulator of IFN responses in human cells and was the first member of the PARP family to be identified as an antiviral factor (Gao et al., 2002). Since then, PARP13 has been recognized to inhibit replication of a broad spectrum of RNA and DNA viruses and to bind single-stranded RNA (ssRNA) (Fig. 4F) (Chiu et al., 2018; Luo et al., 2020). Although PARP13 is versatile and widely effective against various viruses, it is not a universal antiviral protein. The RNA-binding ability of PARP13 highly depends on the CpG content in ssRNA and positively correlates with the amount of CpG dinucleotides in a given RNA sequence (Fros et al., 2021; Gonçalves-Carneiro et al., 2022). However, PARP13 can also recognize cytosine-rich sequences (Gonzalez-Perez et al., 2021), suggesting that multiple sequence features regulate recognition of RNA by PARP13, which could potentially extend its activity to host cellular RNAs (Shao et al., 2024). Although it lacks intrinsic enzymatic activity, PARP13 interacts with numerous cellular factors, including those that modify PARP13 post-translationally as well as cofactors that enhance its antiviral function, such as TRIM25, an E3 ubiquitin and ISG15 ligase (Li et al., 2017). Moreover, PARP13 has been found to interact with viral proteins of the influenza A virus, resulting in their proteasomal degradation (Liu et al., 2015).

PARP13 degrades viral mRNA by recruiting cellular poly(A)-specific ribonuclease (PARN) to shorten the 3′ poly(A) tail, as well as components of RNA exosomes to stimulate 3′-to-5′ RNA degradation (Chiu et al., 2018; Guo et al., 2007; Zhu et al., 2011). Different DEAD-box helicases (e.g. DDX17, DHX30) bind to PARP13 and function to unwind viral RNA, promoting its degradation via exosome-mediated pathways (Fig. 4F) (Chen et al., 2008; Ye et al., 2010). In the context of SARS-CoV2 coronavirus, PARP13 directly interacts with the viral RNA, impairs frameshifting and inhibits viral replication (Zimmer et al., 2021). Besides facilitating degradation of viral RNA, PARP13 inhibits the translation of target mRNAs by disrupting the interaction between eIF4G and eIF4A translational initiation factors, while also facilitating mRNA degradation (Fig. 4F) (Zimmer et al., 2021).

In addition, PARP13 enhances host innate immunity to restrict viral infections by synergizing with the RIG-I signaling pathway. RIG-I is a cytoplasmic PRR possessing ATP-dependent DExD/H box RNA helicase activity that detects short dsRNA and 5′-triphosphate RNA, which triggers IFN-I production (Min et al., 2020). The short isoform of PARP13 associates with RIG-I to promote its oligomerization and ATPase activity, which lead to robust activation of the transcription factors IRF3 and NF-κB (Hayakawa et al., 2011). In this pathway, TRIM25 and another E3 ubiquitin ligase, RIPLET (also known as RNF135), have been shown to act as PARP13 cofactors, although the involvement of TRIM25 remains debated (Choudhury et al., 2022; Hayman et al., 2019). RIPLET ubiquitylates RIG-I bound to dsRNA, stabilizing RIG-I oligomerization, which activates mitochondrial antiviral signaling protein (MAVS) and stimulates IFN-I production (Buckmaster and Goff, 2022). An additional PARP13 interactor is oligoadenylate synthetase 3 (OAS3), which colocalizes to stress granules with PARP13 and echovirus E7 RNA (Odon et al., 2019; Goonawardane et al., 2021). OAS3 plays a key role in blocking viral replication by activating the latent ribonuclease (RNase L) (Schwartz and Conn, 2019); by degrading viral RNA and inhibiting global protein synthesis, RNase L promotes an antiviral state. This suggests cross talk with PARP13 in coordinating viral degradation and innate immune responses against virus infection.

Lastly, like the other PARP enzymes discussed above, PARP14 is upregulated by IFNs (Heer et al., 2020). PARP14 is essential for increased IFN-I production in response to infection by coronaviruses that lack ADP-ribosylhydrolase activity – an enzymatic function that reverses host-mediated ADP-ribosylation – or to stimulation with the RNA-mimicking molecule polyinosinic:polycytidylic acid [poly(I:C)] (Grunewald et al., 2019; Parthasarathy and Fehr, 2022). Of note, PARP14 is responsible for the increased MARylation observed upon transfection with poly(I:C), indicating a prominent role in IFN signaling (Kar et al., 2024; Ribeiro et al., 2024). Recent studies have assessed the role of PARP14 in infections by various viruses, including herpes simplex virus 1 (HSV-1) and several negative-sense RNA viruses, such as VSV, Ebola virus and Nipah virus (Parthasarathy et al., 2024 preprint). However, PARP14 catalytic activity is not always required for its antiviral effects, suggesting that it might have multiple mechanisms of action (Parthasarathy et al., 2024 preprint).

## MARylating PARPs in cancer

**Fig. 5.**
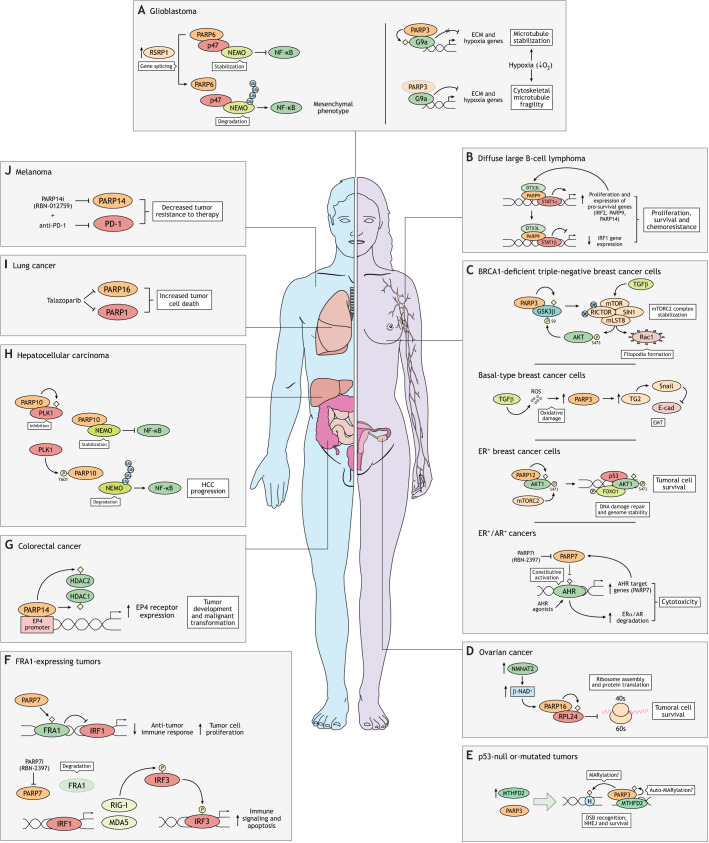
**MARylating PARPs in cancer.** MARylating PARPs play key roles in the onset and progression of cancer by influencing essential cellular processes, affecting cancer cell survival, proliferation and resistance to therapy through multiple oncogenic and tumor suppressive pathways. (A) In glioblastoma cells, PARP3 regulates G9a-mediated transcriptional repression and affects microtubule stability, and a truncated form of PARP6 enhances NF-κB activation, promoting a mesenchymal phenotype. (B) In aggressive DLBCL, PARP9 promotes tumor growth and chemoresistance by suppressing the tumor suppressor IRF1 and upregulating the proto-oncogene IRF2, thereby inhibiting the IFNγ–STAT1–IRF1–p53 axis through the regulation of both STAT1α and the antagonistic isoform STAT1β. (C) In BRCA1-deficient TNBC cells (estrogen receptor, progesterone receptor and HER2 negative), PARP3 regulates TGFβ-induced cytoskeletal rearrangements and lamellipodia formation, and its inhibition is selectively lethal. In basal-type breast cancer cells (mesenchymal-like), augmented levels of PARP3, stimulated by TGFβ, induce the expression of TG2 and the transcription factor Snail, inhibiting E-cad and enhancing EMT. In estrogen receptor-positive (ER^+^) cells, PARP12 promotes tumor cell survival by activating AKT1 kinase through MARylation. In these cells, PARP12 suppression increases DNA damage, impairs AKT1 kinase function and activates the apoptotic cascade via FOXO1. In breast cancers positive for both estrogen receptor and androgen receptor (ER^+^/AR^+^), a combination of PARP7 inhibitor and AHR agonists is a promising therapeutic strategy to treat hormone-refractory tumors. (D) PARP16 promotes ovarian cancer cell growth and survival by MARylating ribosomal proteins, allowing the cells to manage proteotoxic stress and avoid excessive protein aggregation. (E) In p53-mutated tumors, PARP3 interacts with MTHFD2 to enhance DNA repair through NHEJ and support cell survival. (F) In FRA1-expressing tumors, PARP7 inhibition reduces cell proliferation and survival by activating immune signaling and pro-apoptotic genes. (G) PARP14 promotes tumorigenesis in CRC by MARylating HDAC1 and HDAC2 to activate EP4 expression, stimulating cell proliferation, invasion and metastasis. PARP14 inhibition suppresses tumor growth. (H) In HCC, PARP10 regulates NF-κB p65 by interacting with NEMO and PLK1. Elevated PLK1 levels inhibit PARP10, activating the NF-κB pathway to promote tumor growth and metastasis while reinforcing a negative feedback loop. (I) In SCLC, talazoparib, which targets both PARP1 and PARP16, enhances the effect of PARP1 inhibition, making PARP16 a promising therapeutic target for lung and other cancers. (J) PARP14 contributes to immune checkpoint blockade therapy resistance in melanoma by enhancing IFNγ signaling. PARP14 inhibition could potentially improve patient survival by reshaping immune responses. ECM, extracellular matrix; ERα, estrogen receptor α; H, histone; MDA5, melanoma differentiation-associated protein 5, also known as IFIH1; *P*, phosphorylation; PARP7i, PARP7 inhibitor; PARP14i, PARP14 inhibitor; p47, NSFL1 cofactor p47; SIN1, stress-activated MAPK-interacting protein 1, also known as MAPKAP1; Ub, ubiquitin.

Dysregulation of MARylating PARPs has been implicated in various cancers across different tissues. Notably, the development of specific PARP inhibitors has advanced our understanding of MARylating PARP function and biology and has highlighted their potential as pharmacological targets in cancer treatment. Below, we discuss examples of MARylating PARPs – namely PARP3, PARP6, PARP7, PARP9–12, PARP14 and PARP16 – that have been linked to cancer progression and survival (Fig. 5A–J).

In breast cancer cell lines, PARP3 expression correlates with mesenchymal cell phenotype (Fig. 5C). PARP3 expression increases in response to reactive oxygen species (ROS) generated by TGFβ signaling, a recognized driver of epithelial-to-mesenchymal transition (EMT). During EMT, cells remodel cell–cell and cell–extracellular matrix interactions, inducing the detachment of epithelial cells from the basement membrane. In cancer, this transition enhances the tumor-initiating and metastatic potential of tumoral cells (Dongre and Weinberg, 2019). TGFβ-stimulated PARP3 induces the expression of transglutaminase 2 (TG2, also known as TGM2) and the transcription factor Snail (also known as SNAI1), resulting in inhibition of E-cad, enhancing EMT (Karicheva et al., 2016). Moreover, PARP3 inhibition shows selective lethality in BRCA1-deficient triple-negative breast cancer (TNBC) cells compared to BRCA1-proficient cells. PARP3 regulates TGFβ-induced cytoskeletal rearrangement, leading to formation of lamellipodia, the numbers of which are elevated in BRCA1-deficient breast cancer cells (Beck et al., 2019). In this phenomenon, PARP3 inhibits GSK3β-mediated degradation of Rictor, a component of the mTORC2 kinase complex. Rictor stabilizes the mTORC2 complex, which is in parallel activated by TGFβ. This results in both stimulation of Rac1, which promotes formation of filopodia, and phosphorylation of AKT kinases on Ser473, which inhibits GSK3β, reinforcing a feedback loop (Beck et al., 2019).

PARP3 also exerts a role in p53 (TP53)-mutated tumors (Fig. 5E). Transcription of the mitochondrial bifunctional enzyme MTHFD2 is repressed by p53, and p53 mutation or deletion thus causes upregulation of MTFHD2, enhancing both one-carbon metabolic flux to purines and DNA repair via NHEJ, which promotes cell proliferation and survival (Li et al., 2021b). A nucleus-localized pool of MTHFD2 directly modulates the DDR through binding and catalytic activation of PARP3. DNA damage induced by MTHFD2 depletion might result from defects in NHEJ, as PARP3 overexpression largely reverses DNA damage in MTHFD2-depleted cells (Li et al., 2021b).

In glioblastoma cells, PARP3 has been identified as a novel interactor and regulator of the histone methyltransferase G9a (also known as EHMT2; Fig. 5A) (Nguekeu-Zebaze et al., 2022). MARylation by PARP3 modulates G9a-mediated transcriptional repression of genes involved in cell adhesion and hypoxia response. Additionally, PARP3 dysfunction weakens cytoskeletal microtubules, increasing their sensitivity to microtubule-destabilizing agents, in part through PARP3- and G9a-mediated signaling. This suggests that PARP3-specific inhibitors in combination with microtubule-targeting agents could represent a potential therapeutic approach for glioblastoma treatment (Nguekeu-Zebaze et al., 2022).

High levels of PARP6 inversely correlate with tumor growth and are associated with good prognosis in colorectal carcinoma (CRC) (Tuncel et al., 2012). PARP6 suppression, either by the PARP inhibitor AZ0108 or small interfering RNA (siRNA)-mediated knockdown, promotes an anti-tumor response in a panel of breast cancer lines as well as in breast cancer xenografts, likely through the MARylation of CHK1 during mitosis (Wang et al., 2018). In glioblastoma, arginine/serine-rich protein 1 (RSRP1) catalyzes PARP6 exon 18 skipping, generating the truncated oncogenic form of PARP6 (PARP6-s), which lacks the catalytic triad of residues essential for MARylating activity (Fig. 5A). PARP6-s expression enhances NF-κB activation, ultimately promoting the glioblastoma mesenchymal phenotype (Li et al., 2021c).

PARP7, also known as 2,3,7,8-tetrachlorodibenzo-*p*-dioxin (TCDD)-inducible PARP (TiPARP), has been identified both as a target gene and a regulator of various nuclear transcription factors that are activated by hormones or environmental cues, including AHR (Diani-Moore et al., 2010; Rijo et al., 2021), estrogen receptor (Rasmussen et al., 2021), androgen receptor (AR) (Kamata et al., 2021a,b; Yang et al., 2021), hypoxia-inducible factor 1α (HIF1α) (Zhang et al., 2020) and liver X receptors (LXRs) (Bindesbøll et al., 2016). PARP7 modulates the transcriptional activity and protein stability of these factors through MARylation. As mentioned above, PARP7 serves as a crucial regulator of innate immune signaling by repressing the IFN-I response, a pathway often altered in cancer (Gozgit et al., 2021; Sanderson et al., 2023). In cancer cells, genomic instability leads to the accumulation of cytosolic nucleic acids, activating the cyclic GMP-AMP synthase–stimulator of interferon genes (cGAS–STING) pathway and the expression and secretion of IFN-I. PARP7 plays a pivotal role in enabling immune evasion by suppressing the IFN-I response (Manetsch and Hottiger, 2025). In lung and breast cancer cells, PARP7 loss-of-function reduces cell proliferation and survival through downregulation of the transcription factor FRA1 (also known as FOSL1), which inhibits the interferon regulatory factors IRF1 and IRF3. This leads to the upregulation of IRF1-and IRF3-dependent pro-inflammatory and pro-apoptotic genes, driving apoptosis (Fig. 5F) (Manetsch et al., 2023).

However, the role of PARP7 appears to vary across cancer types. In prostate cancer, PARP7 acts as a positive regulator of the AR complex, enhancing receptor MARylation, nuclear translocation and transcription of its target genes (Kamata et al., 2021a,b; Yang et al., 2021). In ovarian cancer, PARP7 MARylates α-tubulin, promoting cancer cell migration; consequently, PARP7 inhibition reduces cell growth and motility in ovarian, cervical and kidney cancer cells (Palavalli Parsons et al., 2021). In contrast, the results of studies in breast cancer cells suggest that PARP7 negatively regulates estrogen receptor α (ESR1) signaling (Rasmussen et al., 2021). PARP7 knockdown promotes tumor growth in MCF-7 human breast cancer cell xenografts lacking immune cells or a functional immune system, which could potentially explain these divergent outcomes (Rasmussen et al., 2023). Recent studies in EO771 mouse mammary cancer cells and syngeneic tumor models have revealed that PARP7 loss modestly decreases cell proliferation in immunodeficient mice (Rasmussen et al., 2023). However, in immunocompetent mice, PARP7 loss significantly reduces tumor growth, particularly in mutant Parp7 H532A mice, by enhancing IFN-I signaling and CD8-positive T cell infiltration (Rasmussen et al., 2023). The efficacy of PARP7 inhibitors in promoting tumor regression therefore depends on an intact IFN-I pathway and robust immune responses. The only PARP7 inhibitor currently in clinical trials, RBN-2397, is being tested for treating non-small cell lung cancer (NSCLC) in combination with pembrolizumab (clinical trial ID NCT05127590; https://clinicaltrials.gov/study/NCT05127590), a monoclonal antibody that blocks the PD-1 immune checkpoint receptor (also known as PDCD1), thereby enhancing T cell-mediated immune responses against tumors. Thus, targeting PARP7 activity in immune cells holds significant promise for strengthening anti-tumor immunity (Brooks et al., 2023).

In this context, to overcome resistance to immune checkpoint inhibitors, a novel bifunctional conjugate targeting both programmed death-ligand 1 (PD-L1, also known as CD247) and PARP7 has been synthesized. This compound has been found to enhance Jurkat T cell cytotoxicity against MDA-MB-231 human breast cancer cells in a concentration-dependent manner in a co-culture assay. Moreover, it exhibits significant anti-tumor efficacy in a B16-F10 mouse melanoma model, likely by activating the immune microenvironment, as shown by immunohistochemical analysis (Gao et al., 2024). Although still at an early stage, these findings suggest that this dual-target compound is a promising lead for future PD-L1-based multi-target immunotherapy development. In addition, a recent study highlights the potential of combining PARP7 inhibitors with AHR agonists as a therapeutic strategy for tumors that no longer respond to hormone-based therapies (Fig. 5C). This treatment combination induces cytotoxicity in cancer cells that are unresponsive to either agent alone, including in breast and prostate cancer models resistant to hormone therapies. Mechanistically, PARP7 suppresses AHR transactivation through ADP-ribosylation, and PARP7 inhibitors block this process, leading to sustained AHR activation. AHR agonists alone promote AHR activity but also trigger a negative feedback loop that limits prolonged activation. When combined, AHR agonists maintain AHR nuclear localization and activation while PARP7 inhibitors prevent AHR deactivation, ultimately leading to cytotoxic effects and degradation of aryl hydrocarbons and estrogen receptors, including their treatment-resistant mutants (Chen et al., 2022, 2025).

PARP9, originally known as B-aggressive lymphoma-1 (BAL1), was identified as a risk-associated gene in aggressive diffuse large B-cell lymphoma (DLBCL) (Aguiar et al., 2000, 2005). PARP9 is constitutively expressed in high-risk DLBCLs characterized by an active inflammatory response and is strongly linked to IFN-related gene expression. Functionally, PARP9 represses the tumor suppressor IRF1 while upregulating the proto-oncogene IRF2, thereby inhibiting the IFNγ–STAT1–IRF1–p53 signaling axis and promoting proliferation, survival and chemoresistance in DLBCL (Fig. 5B) (Camicia et al., 2013).

PARP9 also acts as a survival factor in metastatic prostate cancer, head and neck squamous cell carcinoma (HNSCC), and breast cancer in cooperation with DTX3L and PARP14 (see below). In prostate cancer, this complex drives proliferation, chemoresistance and survival in a manner dependent on PARP14 catalytic activity. Specifically, PARP9 and DTX3L repress IRF1, thereby inhibiting apoptosis while promoting pro-survival signals (Bachmann et al., 2014). The PARP9–DTX3L complex also modulates AR activity and expression of its target genes, further supporting its oncogenic role (Yang et al., 2021). In HNSCC, PARP9, DTX3L and PARP14 protein levels significantly correlate. Mechanistically, the PARP9–DTX3L complex post-transcriptionally regulates PARP14, contributing to tumor cell survival. In HNSCC and HeLa cell lines, depletion of PARP9, DTX3L or PARP14 results in reduced proliferation and increased apoptosis, ultimately impairing cell survival (Saleh et al., 2024). PARP9 is also implicated in breast cancer progression (Hong et al., 2024; Tang et al., 2018), particularly in tumor immune evasion. In fulvestrant-resistant breast cancer cells, PARP9 depletion disrupts the PI3K–AKT pathway, leading to PD-L1 downregulation and a reduction in both chemoresistance and immune escape (Hong et al., 2024). PARP9 is also highly expressed in gastric cancer, where it promotes the malignant behavior of gastric cancer cells. PARP9 overexpression correlates with poor patient prognosis (Li et al., 2024). Mechanistic studies indicate that PARP9 interacts with SOX6, a key regulator of tumor progression, to enhance resistance to apoptosis and DNA damage, further supporting its contribution to gastric cancer pathogenesis (Li et al., 2024).

PARP10 was originally identified as an interactor of the transcription factor and oncogene c-MYC and was proposed to be a regulator of cell proliferation (Yu et al., 2005). Nucleus-localized PARP10 can confer an advantage in the growth of transformed cells by alleviating replicative stress through its role in TLS, thus improving resilience to DNA damage (Khatib et al., 2024a; Nicolae et al., 2014; Schleicher et al., 2018). Furthermore, PARP10 is involved in the regulation of the NF-κB subunit p65 (also known as RELA) through interactions with NF-κB essential modulator (NEMO, also known as IKBKG) (Verheugd et al., 2013). PARP10 also cross-regulates its interactor polo-like kinase 1 (PLK1) (Feijs et al., 2013). In hepatocellular carcinoma (HCC), elevated levels of PLK1 inhibit PARP10 activity, which in turn, allows activation of the NF-κB pathway through NEMO. Nuclear NF-κB stimulates the transcription of multiple target genes promoting HCC growth and metastasis while repressing the transcription of PARP10, reinforcing a negative feedback loop (Fig. 5H) (Tian et al., 2020). In non-synchronized cells, the depletion of PARP10 stimulates AURKA-dependent phosphorylation of AKT kinases on Ser473, inducing EMT (Zhao et al., 2018). Collectively, evidence suggests that the downregulation of PARP10 could be a key factor in the development of the HCC phenotype and in HCC metastatic potential. Conversely, in oral squamous cell carcinoma (OSCC), upregulation of PARP10 correlates with poor prognosis. Studies in OSCC cell lines and tissues suggest that this is most likely due to the activation of PI3K–AKT and mitogen-activated protein kinase (MAPK) signaling pathways by PARP10, which would explain the enhancement in cell proliferation, apoptosis and metastatic capability observed for this type of tumor (Zhou et al., 2022).

In solid tumors, resistance to therapy and evasion of anti-tumor immunity are aided by an immune suppressive tumor microenvironment (TME). PARP11 acts as a key regulator of the immune suppressive TME. Soluble factors released by the TME increase PARP11 expression in cytotoxic T cells (CTLs), leading to degradation of IFNAR1 and inactivation of intratumoral CTLs. Importantly, pharmacological targeting of PARP11 represents a valid strategy to improve the efficacy of chimeric antigen receptor T cell (CAR-T) therapy (Zhang et al., 2022).

PARP12 has also been implicated in cancer biology, although its specific contribution remains only partially understood. Accordingly, patients with higher expression of PARP12 and ADP-ribosylation-related genes ARL8B, ARFIP2 and ADPRHL1 benefit more from immune checkpoint inhibitor treatment, suggesting a potential role of ADP-ribosylation in HCC biology (Jiang et al., 2024). In the context of breast cancer, PARP12 has been identified as an active participant in the IFN–STAT1 signaling pathway, which promotes survival and regrowth of breast cancer cells after chemotherapy (Gaston et al., 2016). Additionally, recent findings indicate that PARP12 mediates MARylation of AKT1 kinase, leading to its activation and subsequently favoring cell survival. Transcriptional suppression of PARP12 increases DNA damage, under which conditions AKT kinases are functionally impaired in targeting the downstream effector FOXO1, resulting in elevated FOXO1 protein levels and activation of the apoptotic cascade, ultimately promoting cell death (Fig. 5C) (Pavithran et al., 2025). Although the molecular mechanisms underpinning the role of PARP12 in breast cancer are still being elucidated, these findings highlight its potential as a promising therapeutic target in breast cancer treatment.

PARP14 has been implicated in cancer progression across multiple tumor types. In CRC, PARP14-mediated MARylation of histone deacetylases HDAC2 and HDAC3 facilitates the expression of EP4 (also known as PTGER4), a prostaglandin receptor frequently upregulated in the TME. EP4 plays a crucial role in promoting cell proliferation, invasion and metastasis. Inhibiting PARP14 effectively suppresses EP4 receptor activation, reducing tumor cell proliferation (Fig. 5G) (Mashimo et al., 2022). Beyond CRC, PARP14 contributes to tumorigenesis in B-cell lymphoma by inhibiting JNK1 (MAPK8) signaling, thereby preventing apoptosis, and in acute myeloid leukemia by enhancing glycolysis to support cancer cell proliferation (Barbarulo et al., 2013). Consistent with its role in maintenance of genome integrity, a synthetic lethal interaction (where combined inhibition of two pathways is lethal to cells but inhibition of either alone is not) has been identified between PARP14 and the ATR–CHK1 pathway. PARP14 loss sensitizes cells to ATR–CHK1 inhibition, revealing a potential therapeutic vulnerability (Dhoonmoon et al., 2020).

Additionally, elevated PARP14 protein levels have been observed in HNSCC and other malignancies, often in conjunction with its interacting partners PARP9 and DTX3L. As mentioned above, depleting PARP14, PARP9 or DTX3L significantly reduces the survival and proliferation of HeLa and HNSCC cells (Saleh et al., 2024). This effect is independent of PARP14 catalytic activity but requires a specific C-terminal domain essential for forming the PARP14–PARP9–DTX3L heterotrimer. Mechanistically, this complex inhibits PARP14 auto-MARylation, thereby stabilizing PARP14 protein levels and sustaining pro-survival signaling (Saleh et al., 2024).

Intriguingly, PARP14 also plays a key role in immune evasion. Melanoma tumors that acquire resistance to immune checkpoint blockade therapy, such as anti-PD-1 immunotherapy, exhibit elevated PARP14 mRNA levels alongside increased IFNγ gene expression (Wong et al., 2023). Tumors that spontaneously relapse in immunocompetent female mice after anti-PD-1 therapy exhibit upregulated IFNγ signaling; however, these tumors can be re-sensitized through PARP14 inhibitor treatment, highlighting PARP14 as a viable target for overcoming IFNγ-driven resistance to immune checkpoint blockade (Fig. 5J) (Wong et al., 2023). Of note, a recent study has identified a gene signature linked to PARP14 inhibition that correlates with improved patient survival: the combination of PARP14 and PD-1 inhibition reshapes macrophage populations and sustains CTL function by inducing quiescence. This suggests that T cell-induced quiescence could help preserve T cell function during chronic stimulation, potentially enabling prolonged disease control if CTL reactivation is carefully regulated (Leshem et al., 2025).

PARP14 inhibitors might also offer therapeutic benefits in other tumor models. In multiple myeloma, where PARP14 is upregulated and implicated in mitophagy induction, PARP14 inhibition could provide clinical advantages (Zhang et al., 2024). Similarly, in ovarian cancer, PARP14 inhibition suppresses tumor growth by modulating MARylation of receptor for activated C kinase 1 (RACK1) and stress granule formation (Challa et al., 2025).

Lastly, studies on ovarian cancer cells have uncovered a central role of PARP16 in cell survival. Specifically, high levels of cytosolic β-NAD^+^, produced by elevated nicotinamide mononucleotide adenylyltransferase 2 (NMNAT2) expression in ovarian cancer, activate PARP16 on the ER membrane, where PARP16 MARylates ribosomal proteins, including RPL24, impairing polysome assembly and decreasing protein synthesis. This allows ovarian cancer cells to circumvent proteotoxic stress caused by a high rate of protein translation that would otherwise cause an excessive ER load and protein aggregation, resulting in reduced cell growth (Fig. 5D) (Challa et al., 2021). Interestingly, PARP16 is a specific pharmacological co-target of the PARP1 inhibitor talazoparib (Palve et al., 2022). Compared to other PARP1 inhibitors, talazoparib displays higher efficacy in reducing proliferation and inducing cell death in small cell lung cancer (SCLC) cell lines thanks to the parallel inhibition of PARP1 and PARP16, suggesting that the latter is an attractive therapeutic target for the treatment of different types of cancers (Fig. 5I) (Palve et al., 2022).

## Future developments

The steady rise in studies on MARylation over the past 20 years highlights the growing interest in this PTM within the scientific community. This excitement has been driven by identification of various PARP enzymes, advancement of techniques enabling the discovery of MARylation targets across cellular compartments and expanding knowledge of their physiological roles, many of which have direct implications in disease mechanisms. Consequently, a major focus in the field is the development of selective inhibitors for different PARP enzymes. In particular, our increasing understanding of MARylating PARPs in cancer and their role in regulating IFN signaling is fostering the development of new combinatorial therapies targeting both PARP enzymes and components of the immune response. In this context, the PARP7 inhibitor RBN-2397, initially tested as a single agent in solid tumors with inconclusive results (clinical trial ID NCT04053673; https://clinicaltrials.gov/study/NCT04053673), is currently being evaluated in individuals with NSCLC who initially responded to treatment with a PD-1 or PD-L1 inhibitor but later experienced disease progression (clinical trial ID NCT05127590; https://clinicaltrials.gov/study/NCT05127590). This example is indicative of the great potential that novel PARP inhibitors will have as soon as they become available. Indeed, several research groups are working in this direction (Basavaraja et al., 2024; Bejan et al., 2022; Murthy et al., 2023; Wong et al., 2023).

In our view, another crucial area requiring further exploration is the interplay among multiple PARPs in regulating cellular functions. While our understanding of this cooperation is emerging in the context of host antiviral responses (Kerr et al., 2023; Ryan et al., 2025), similar interactions are expected to occur in other cellular processes. Many MARylating PARPs modulate the IFN pathway or DDR, thereby playing key roles in cell survival and tumor progression. Indeed, cross talk between PARP1 and PARP12 has already been demonstrated in the cellular response to oxidative stress (Catara et al., 2017). Currently, the available data on these interactions remain fragmented, underscoring the need for a more comprehensive analysis in order to construct a map of the PARP network. Identifying crucial regulatory hubs controlled by multiple PARPs will provide a deeper understanding of MARylation-driven biology, ultimately leading to more effective pharmacological interventions.

Finally, how distinct functions are regulated by the same PARP and, conversely, how the same function is executed under different cellular conditions are important questions for further investigation. PARP14 and PARP12 have been implicated in diverse processes including antiviral defense, DNA repair and membrane trafficking. Whether these functions are context dependent (for example, varying with cell type, developmental stage or stimulus) or mechanistically interconnected remains unclear. For example, the trafficking role of PARP12 might support its antiviral activity, and PARP14 might switch between immune signaling and DNA repair depending on cellular context or regulatory cues. Dissecting these dynamics will be key to understanding PARP functional versatility.
